# Structural Studies of the Lipopolysaccharide Isolated from *Plesiomonas shigelloides* O22:H3 (CNCTC 90/89)

**DOI:** 10.3390/ijms21186788

**Published:** 2020-09-16

**Authors:** Anna Maciejewska, Brygida Bednarczyk, Czeslaw Lugowski, Jolanta Lukasiewicz

**Affiliations:** Laboratory of Microbial Immunochemistry and Vaccines, Ludwik Hirszfeld Institute of Immunology and Experimental Therapy, Polish Academy of Sciences, Weigla 12, 53-114 Wroclaw, Poland; brygida.bednarczyk6@gmail.com (B.B.); czeslaw.lugowski@hirszfeld.pl (C.L.); jolanta.lukasiewicz@hirszfeld.pl (J.L.)

**Keywords:** lipopolysaccharide, LPS, core oligosaccharide, O-specific polysaccharide, NMR, mass spectrometry

## Abstract

*Plesiomonas shigelloides* is a Gram-negative, rod-shaped bacterium which causes foodborne intestinal infections, including gastroenteritis. It is one of the most frequent causes of travellers’ diarrhoea. Lipopolysaccharide (LPS, endotoxin), an important virulence factor of the species, is in most cases characterised by a smooth character, demonstrated by the presence of all regions, such as lipid A, core oligosaccharide, and O-specific polysaccharide, where the latter part determines O-serotype. *P. shigelloides* LPS is still a poorly characterised virulence factor considering a “translation” of the particular O-serotype into chemical structure. To date, LPS structure has only been elucidated for 15 strains out of 102 O-serotypes. Structures of the new O-specific polysaccharide and core oligosaccharide of *P. shigelloides* from the Czechoslovak National Collection of Type Cultures CNCTC 90/89 LPS (O22), investigated by chemical analysis, mass spectrometry, and ^1^H,^13^C nuclear magnetic resonance (NMR) spectroscopy, have now been reported. The pentasaccharide repeating unit of the O-specific polysaccharide is built of one d-Qui*p*NAc and is rich in four d-Gal*p*NAcAN residues. Moreover, the new core oligosaccharide shares common features of other *P. shigelloides* endotoxins, i.e., the lack of phosphate groups and the presence of uronic acids.

## 1. Introduction

*Plesiomonas shigelloides* is the only species in the genus *Plesiomonas* and belongs to the *Enterobacteriaceae* family. It is usually isolated from fish, crabs, prawns, mussels, and oysters, as well as birds, amphibians, and mammals. The species is a Gram-negative, oxidise-positive, rod-shaped bacterium which causes intestinal infections associated with consumption of seafood, uncooked food, and contaminated water. Foreign travel, particularly to Latin America, the Caribbean, and South and Southeast Asia, is a second major risk factor associated with *Plesiomonas* infections in humans [1]. Recently, the species was pointed out as an emerging agent of gastroenteritis that varies from a secretory or watery diarrhoea to more severe dysentery-like symptoms [2]. *P. shigelloides* infection used to be omitted during diagnostics, since it occurred as a concurrent infection with well-established stool pathogens such as *Salmonella* spp., *Campylobacter jejuni*, *Shigella* spp., or *Vibrio* spp. [2]. Extra-intestinal infections caused by *P. shigelloides*, most notably, meningitidis in neonates, bacteraemia, sepsis, and septic shock, were uncommonly reported for this, but sepsis and meningitidis are related with the serious course and high fatality rate [3,4].

The pathogenicity of *P. shigelloides* is poorly understood. Lately, a repeats-in-toxin (RTX) cytolysin, lysophospholipase, a twin-arginine translocation (Tat) system, and the antibacterial type VI secretion effector phospholipase A1 were described [5] among known *P. shigelloides* virulence factors, such as cholera-like toxin [6], thermostable and thermolabile toxins [7], β-haemolysin [8], and a cytotoxin complex containing lipopolysaccharide (LPS, endotoxin), which causes apoptosis of infected host cells [9]. Despite the identification of virulence factors, so far, no consistent pathogenicity mechanism has been described in detail based on experimental evidence. It was demonstrated that these bacteria adhere to and enter epithelial human cell line Caco-2 [1,10]. *P. shigelloides* are able to occupy the cytosol and migrate from the cytoplasmic vacuoles [10]. LPS, the main constituent of the outer membrane of the cell envelope, triggers the immune response via Toll-like receptor. LPS is essential for function and survival of Gram-negative bacteria, covering about 70% of the surface of the bacterial cell, and protects against defence mechanisms of infected macro-organisms. It plays an important role as a virulence factor in cases of sepsis and septic shock. LPS is a thermo-stable O antigen. It consists of lipid A, core oligosaccharide, and O-specific polysaccharide built up of oligosaccharide repeating units, where the latter part determines O-serotype. The polysaccharide part determines the serological specificity used to distinguish and identify serotypes of Gram-negative bacteria.

Despite the rising knowledge of *P. shigelloides* LPS structures over the past two decades, this virulence factor is still poorly characterised considering a relation between a particular O-serotype and the chemical structure. To date, complete or partial LPS structures have been elucidated only for 15 strains out of 102 identified O-serotypes [11]. Structures of the 13 O-specific polysaccharides have been elucidated to date for strains 22074, 12254 [12], and AM36565 [13], and serotypes O51 [14], O17 [15,16], O1 [17,18,19], O33 [20], O24 [21], O12 [22], O54 [23], O74 [24], O37 [25], and O36 [26]. The core oligosaccharides were elucidated for strains assigned to serotypes O13 [27], O17 [15,16], O1 [18,19], O33 [20], O24 [21], O12 [22], O54 [28], O74 [24], O37 [25], and O36 [26], including its linkage to the O-specific polysaccharide for most of them. The lipid A region, as a part of the complete LPS structure, was elucidated for O54 [29], O74 [30], O37 [25], and O36 [26] serotypes.

The aim of this study was to extend the knowledge about structural features of *P. shigelloides* LPS and to investigate the structure of the new O-specific polysaccharide and core oligosaccharide and the linkage between them of the serotype O22 (strain CNCTC 90/89) LPS. The poly- and oligosaccharides isolated from water phase LPS were investigated by chemical analysis, mass spectrometry, and ^1^H,^13^C nuclear magnetic resonance (NMR) spectroscopy. The pentasaccharide repeating unit of the O-specific polysaccharide of *P. shigelloides* strain CNCTC 90/89, containing the β-d-Qui*p*NAc and rich in four Gal*p*NAcAN residues, has now been described. Furthermore, a new core oligosaccharide is presented that shares *P. shigelloides* common features with the other known *P. shigelloides* cores, that is the lack of phosphate groups and the presence of uronic acids.

## 2. Results

### 2.1. P. Shigelloides O22:H3 (Strain CNCTC 90/89) Showed the Prevailing Smooth–Rough Phenotype

The *P. shigelloides* O22:H3 (strain CNCTC 90/89) LPS was isolated by the phenol/water extraction [31] and purified as previously reported [32]. Both phenol and water phases were collected, however the LPS was obtained only from the water phase (yield 2.86% of dry bacterial mass). The mild acid hydrolysis of the LPS (200 mg) yielded four fractions (1–4) separated by gel filtration on Bio-Gel P-10, with relatively low content of the O-specific polysaccharide (fraction 1). The fraction 1 yielded only 0.89 mg of the O-specific polysaccharide, substituting a core region, contrary to significantly higher amounts of two oligosaccharide fractions, 2 (18.9 mg) and 3 (42.2 mg). Thus, elution profile suggested a smooth–rough phenotype of the analysed LPS.

### 2.2. Structures of the O-Specific Polysaccharide and Core Oligosaccharide

**Figure 1:**
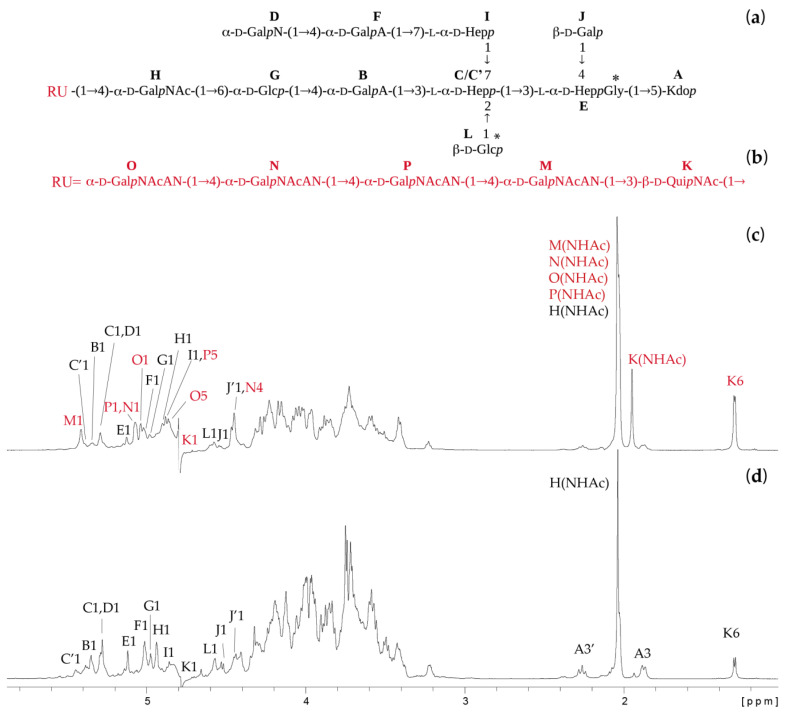
The structures and selected ^1^H NMR spectra of poly- and oligosaccharides isolated from *P. shigelloides* 90/89 LPS. (**a**) The structure of the OS fraction, (**b**) the structure of the first RU (marked in red) of the O-specific polysaccharide linked to the core OS. 1D ^1^H NMR spectra of the (**c**) fraction 2—OSRU and (**d**) fraction 3—OS. The symbol * indicates nonstoichiometric substituents. The capital letters refer to carbohydrate residues, as shown in inset structures and Table 1. The Arabic numerals refer to protons in respective residues. RU stands for the first repeating unit of the O-specific polysaccharide. Letter C’ denotes a residue →2,3,7)-l-α-d-Hep*p*-(1→ instead of the →3,7)-l-α-d-Hep*p*-(1→ (residue C).

Sugar and methylation analyses of the *N*-acetylated fractions combined with determination of the absolute configuration revealed the presence of 2,3,7-trisubstituted l,d-Hep*p*, 3,4-disubstituted l,d-Hep*p*, 7-substituted l,d-Hep*p*, terminal d-Gal*p*N, 6-substituted d-Glc*p*, terminal d-Glc*p*, and terminal d-Gal*p* for fraction 3 and an additional 4-substituted d-Gal*p*NAc and 2-acetamido-2,6-dideoxy-d-glucopyranose (d-Qui*p*NAc, observed only in the absolute configuration analysis) for fraction 2, whereas the last residue was also a constituent of the O-specific polysaccharide (fraction 1). All fractions analysed by NMR spectroscopy and mass spectrometry were finally identified as the O-specific polysaccharide linked to the core OS (fraction 1), the core OS fraction substituted by one repeating unit (RU) of the O-specific polysaccharide (fraction 2—OSRU), and the unsubstituted core OS (fraction 3—OS). Fraction 4 consisted of a mixture of low molecular weight oligosaccharides containing 3-deoxy-D-*manno*-oct-2-ulosonic acid (Kdo) released during mild acid hydrolysis of the LPS (data not shown). An initial NMR analysis of the fraction 1 showed the presence of signals characteristic both for the O-specific polysaccharide (Qui*p*NAc, Gal*p*NAcAN) and the core OS (high-intensity signals), thus it was too heterogenic for a complete NMR analysis (data not shown). The fractions 2 and 3 were analysed by one-dimensional (1D) ^1^H NMR spectra, as shown in Figure 1.

The oligosaccharides OS and OSRU were further analysed using one- and two-dimensional (1D and 2D) ^1^H,^13^C-NMR spectroscopy. All the spin systems were assigned using COSY and TOCSY with different mixing times, and HSQC-DEPT, HSQC-TOCSY, and HMBC spectra. Chemical shift values for the OS and OSRU are shown in Table 1 as average values for the OS residues together with the identified inter-residue connectivities. Chemical shift values (Table 1) were compared with previously published NMR data for respective monosaccharides [18,20,26].

Structural analysis indicated that the core OS (Figure 1a,d and Table 1) is similar to the previously identified core oligosaccharide of *P. shigelloides* strain CNCTC 78/89 [22]. Both structures were undecasaccharides and differed by only one sugar residue in the outer core region. In the strain 90/89, 6-substituted Glc*p* (residue G) was identified instead of the 6-substituted Glc*p*N in the strain 78/89. Since these two core OS structures were very similar, we have focused on the detailed description of sugar residues present in the outer core region only (residues G and H) as a part of the core OS and OSRU fractions. The NMR analysis of the OSRU showed eleven monosaccharides of the core OS (residues A, B, C, D, E, F, G, H, I, J, and L) and an additional five sugars building the RU of the O-specific polysaccharide (residues K, M, N, O, and P) (Figure 1a–c and Table 1).

Residue K with the H-1/C-1 signals at 4.74/102.3 ppm, *J*_C-1,H-1_ ~ 169 Hz, was recognised as the 3-substituted β-d-Qui*p*NAc residue based on the chemical shift of the C-2 signal (55.6 ppm), the downfield shift of the C-3 signal (80.6 ppm), and the signal for an exocyclic CH_3_ group (1.30 ppm, 17.3 ppm). The chemical shift values of the residue K were in good agreement with those previously reported for the 3-substituted β-d-Qui*p*NAc [33].

Residues M, N, and P with the H-1/C-1 signals at 5.41/99.0 ppm (*J*_C-1,H-1_ ~ 179 Hz), 5.07/98.4 ppm (*J*_C-1,H-1_ ~ 178 Hz), and 5.08/98.4 ppm (*J*_C-1,H-1_ ~ 178 Hz) respectively, were assigned as the 4-substituted α- d -Gal*p*NAcAN due to the characteristic signals of C-2 (50.0 ppm), C-6 (174.4, 174.5, and 174.0 ppm), and the downfield shifts of C-4 signals (76.1, 76.3, and 76.0 ppm). The d configuration of all identified 2-acetamido-2-deoxy-galacturonamides (GalNAcAN) was deduced from the ^13^C NMR data using the known regularities in the glycosylation effects [34,35].

Residue O with the H-1/C-1 signals at 5.04/98.6 ppm (*J*_C-1,H-1_ ~ 177 Hz) was assigned as the terminal α-d-Gal*p*NAcAN due to the characteristic signals of C-2 (50.1 ppm) and C-6 (175.1 ppm).

Additionally, the chemical shifts of the H-5/C-5 signals of M, N, P, and O were not pH-sensitive, while the chemical shifts of H-5/C-5 of the galacturonic acids (residue F and B present in the core OS) were shifted with the change of pH from 4.0 to 8.0 (data not shown) as a characteristic feature of uronic acids with free carboxyl group. No pH-dependence suggested that residues M, N, P, and O were primary amides of Gal*p*NAcA. A location of the amino groups at position C-6 of Gal*p*NAcAN residues was further supported by HMBC spectra measured in a 9:1 H_2_O:D_2_O mixture that enabled detection of NH protons (data not shown). Resonances for NH protons were assigned by COSY and TOCSY experiments. The signals at 8.38, 8.34, 8.17, 8.15, 8.10, and 8.07 ppm were assigned as (N2)H of residues K, M, P, N, H, and O, respectively. Furthermore, signals for NH_2_ protons at C-6 of residue M (7.99 and 7.71 ppm), residue N (7.86 and 7.51 ppm), residue P (7.83 and 7.48 ppm), and residue O (7.60 and 7.32 ppm) were also identified.

For the OSRU, the residue H with the H-1/C-1 signals at 4.90/98.0 ppm (*J*_C-1,H-1_ ~ 176 Hz) was assigned as the 4-substituted α-d-Gal*p*NAc based on the characteristic signal of C-2 (50.8 ppm) and the downfield shift of the C-4 signal (76.4 ppm). The presence of the 4-substituted Gal*p*NAc in OSRU instead of the unsubstituted Gal*p*NAc in the OS indicated the linkage between the core oligosaccharide and the O-specific polysaccharide. Moreover, the 4-substituted α-d-Gal*p*NAc was also identified by the OS as a place of substitution by the single terminal β-d-Qui*p*NAc residue (K).

Residue G with the H-1/C-1 signals at δ 4.98/100.9 (*J*_C-1, H-1_ ~ 173 Hz) was recognized as the 6-substituted α-d-Glc*p* based on the large vicinal couplings between all ring protons and the characteristic downfield shift of the C-6 signal (66.0 ppm).

**Figure 2:**
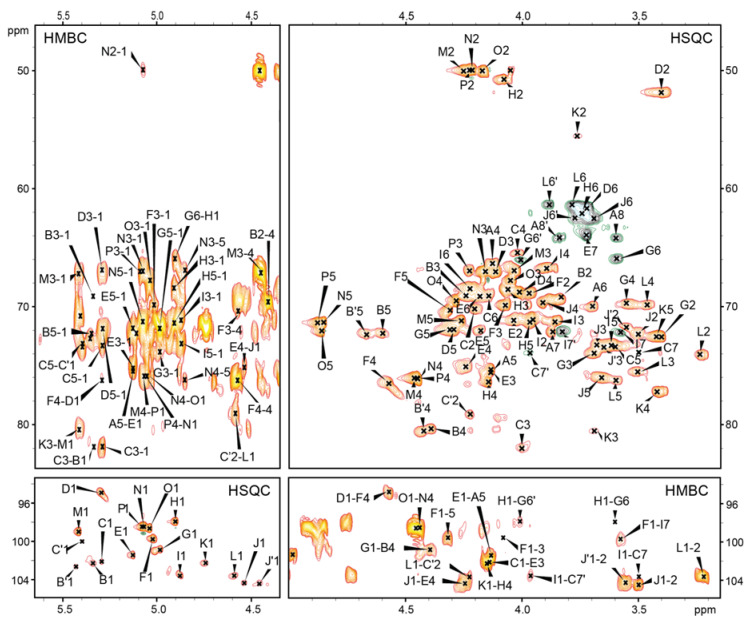
Selected regions of ^1^H,^13^C HSQC-DEPT and HMBC spectra of the OSRU. The capital letters refer to carbohydrate residues, as shown in Figure 1 and Table 1. The Arabic numerals refer to protons and carbons in respective residues. Letter C with a prime sign denotes residues →2,3,7)-l-α-d-Hep*p*-(1→ instead of the →3,7)-l-α-d-Hep*p*-(1→ (C residue). Residues B’ and J’ are variants of residues B and J respectively, present in the core OS containing t-Glc (L residue).

The remaining residues of the outer and inner core region (A, B, C, D, E, F, I, J, and L) have been described in Table 1. The HMBC ^1^H-^31^P correlation NMR spectrum of the OSRU fraction indicated the lack of phosphate groups. The sequence of sugars in the OSRU was identified by HMBC (Figure 2, Table 1) and NOESY (Table 1) experiments, indicating the structure presented in Figure 1a,b. The core OS is substituted by the first RU of the O-specific polysaccharide via →3)-β-d-Qui*p*NAc (residue K) linked to the →4)-α-d-Gal*p*NAc (Residue H).

Moreover, the core OS region revealed some heterogeneity and it was attributed to the nonstoichiometric substitution by glycine (Gly, 26%), the β-d-Glc*p*-(1→ (residue L, 75%) within the inner core region, and a single terminal β-d-Qui*p*NAc (residue K*,* 31%) linked to the residue H, →4)-α-d-Gal*p*NAc-(1→.

### 2.3. Mass Spectrometry Analysis of the Core OS and OSRU Structures and a Glycine Substituent

**Figure 3:**
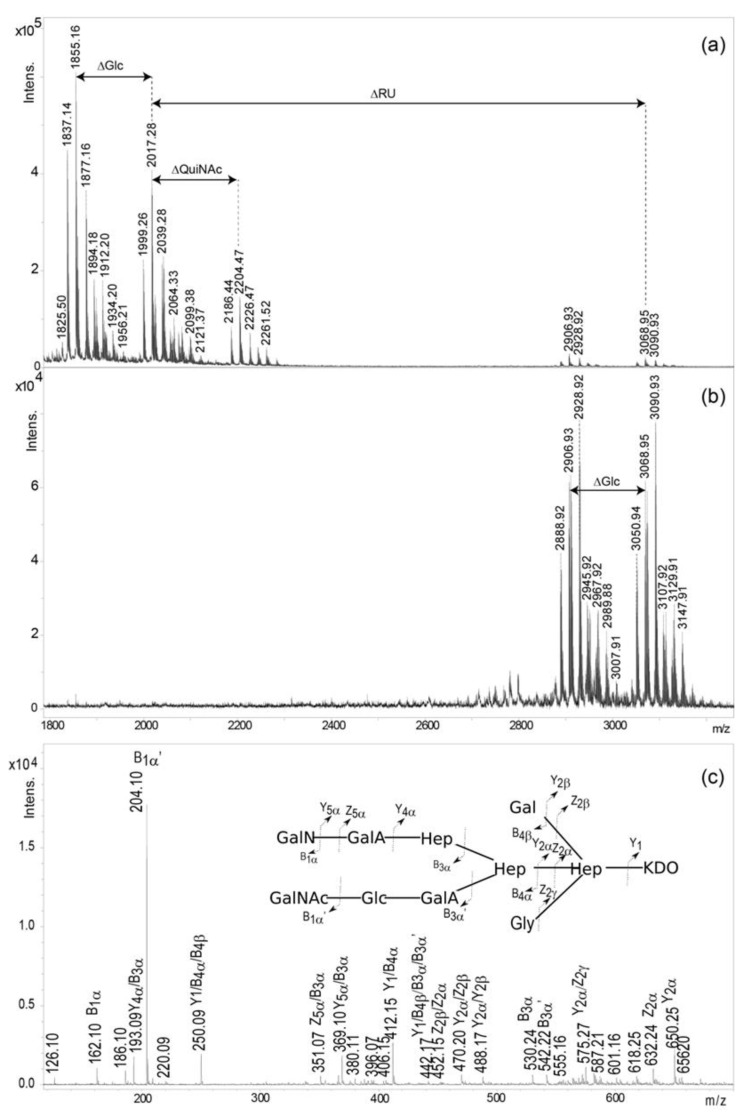
Positive ion mode MALDI-TOF mass spectra of (**a**) the OS, and (**b**) the OSRU fractions isolated from *P. shigelloides* 90/89 LPS. (**c**) The glycine (Gly) substitution identification based on the MS/MS fragmentation of the ion at *m/z* 1912.20 (1+) attributed to the core OS substituted by Gly and accompanied by the OS inset structure explaining interpretation of the fragment ions. The core OS heterogeneity is demonstrated by the presence or the lack of Glc, QuiNAc, and Gly. The fragment ions were presented according to the nomenclature of Domon and Costello [36].

The elucidated structures of the OS and OSRU isolated from *P. shigelloides* CNCTC 90/89 LPS were confirmed by mass spectrometry. The MALDI-TOF mass spectrum of the core OS (Figure 3a) showed ions at *m/z* 2017.28 [M+H]^+^ and *m/z* 1999.26 [M-H_2_O+H]^+^. These ions correspond to three Hep molecules, two Glc, one Gal, two GalA, one GalN, one GalNAc, and one Kdo, which together give a calculated monoisotopic mass of 2016.63 Da, and if Kdo is in the anhydro form, a calculated monoisotopic mass of 1998.62 Da for the undecasaccharide OS.

Ions at *m/z* 1855.16 [M-Hex+H]^+^ and 1837.14 [M-Hex-H_2_O+H]^+^ represent the OS populations devoid of one hexose residue (Glc, residue L). The less abundant ion at *m/z* 2204.47 [M+QuiNAc+H]^+^ represents a glycoform with an additional QuiNAc residue, characteristic for the RU region. Additionally, the presence of the glycine (Gly) in the OS was indicated by ions at *m/z* 1912.20 and 2261.52. Location of Gly was determined by MALDI-TOF tandem mass spectrometry (MS/MS) analysis of the ion at *m/z* 1912.20 (Figure 3c). The MS/MS spectrum consisted of ions at *m/z* 250.09 [Hep-Gly, Y_1_/B_4α_/B_4β_], 412.15 [Hex-Hep-Gly, Y_1_/B_4α_], 442.17 [Hep-Gly-Hep, Y_1_/B_4α_/B_3α_/B_3α’_], 470.20 [Hep-Gly-Kdo, Y_2α_/Z_2β_], and 632.24 [Hex-Hep-Gly-Kdo, Z_2α_], and indicated the location of Gly on 3,4)-Hep.

The mass spectrum of the isolated OSRU component (Figure 3b) showed the main ion at *m/z* 3068.95 [M+H]^+^. This ion corresponding to the hexadecasaccharide structure included one pentasaccharide repeating unit (1051.38 Da) linked to the core oligosaccharide. The ion at *m/z* 2906.93 [M-Hex+H]^+^ corresponded to a structure devoid of the one hexose (Glc). A nonstoichiometric inhibition by Gly was also detected. An interpretation of observed ions is presented in Table 2.

**Figure 4:**
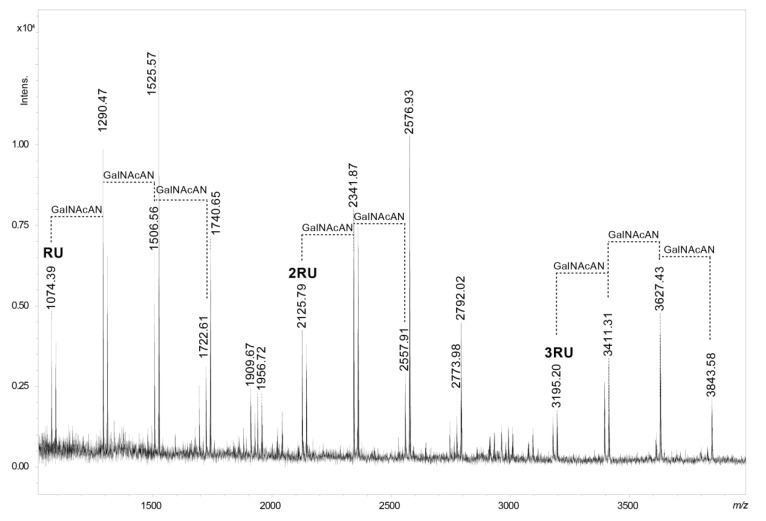
MALDI-TOF mass spectrum in positive ion mode of the partially hydrolysed O-specific polysaccharide (fraction 1) isolated from LPS of *P. shigelloides* 90/89. The *m/z* values represent single charged ions. The RU symbol stands for the one repeating unit of the O-specific polysaccharide, as shown in Figure 1b. Mass differences of GalNAcAN are indicated in the spectrum.

The presence of the core OS glycoforms containing a single QuiNAc and the lack of NMR support for fraction 1 called into question this sugar residue as an integral part of the O-specific polysaccharide RU. Thus, a partial hydrolysis of the O-specific polysaccharide combined with MALDI-TOF MS analysis was used to confirm the pentasaccharide structure of the RU (Figure 4 and Table 3). 

The MALDI-TOF spectrum showed that the ion at *m/z* 1074.39 [M-H_2_O+H, Na]^+^ corresponded to four GalNAcAN molecules and one QuiNAc as components of the RU. It was accompanied by ions of higher *m/z* values (*m/z* 2125.79 and 3195.20). These ions indicated fragments built of two and three RUs respectively, proving that QuiNAc is an integral component of the O-specific polysaccharide. Furthermore, fragments attributed to 2RU and 3RU characterised by similar heterogeneity patterns were also identified.

## 3. Discussion

Lipopolysaccharides of *P. shigelloides* play an important role in the pathogenesis of infections as pathogen-associated molecular patterns triggering toll-like receptor 4 and components of a cytotoxin complex. These virulence factors of *P. shigelloides* are still poorly characterized macromolecules. To date, only 15 structures of *Plesiomonas* LPSs out of 102 O-serotypes have been reported. Although, for some *Plesiomonas* strains, LPSs were extracted [14,17,20,24,25] both from phenol and water phases during phenol-water extraction, and the analysed LPS of *P. shigelloides* 90/89 was recovered only from the water phase.

The analysed LPS showed the prevailing smooth–rough phenotype, since only a trace amount of the O-specific polysaccharide fraction was isolated among poly- and oligosaccharides obtained after mild acid hydrolysis of LPS. Due to this limitation and the heterogeneity of fraction 1, only the first RU structure was elucidated as the pentasaccharide composed of the β-d-Qui*p*NAc and four α-d-Gal*p*NAcAN residues (Figure 1b). A rare monosaccharide, Gal*p*NAcAN, has been found previously in the structure of the O-antigen of *Francisella novicid*a (U112) [37], *F. tularensis* [38], *Escherichia coli* O35 [39] and O121 [40], *Pseudomonas aeruginosa* O6 [41], Pseudomonas chlororaphis subsp. aureofaciens UCM B-306 [42], and *Shigella dysenteriae* type 7 [34]. Amidation of the carboxyl group of hexuronic acids, including d-GalNAcA, has been reported as a feature involved in an adjustment of the optimal charge of the cell surface [42]. The elucidated RU structure constitutes a single biological repeating unit linked to the core OS by the →3)-β- d -Qui*p*NAc substituting the outer core →4)-α-d-Gal*p*NAc. Due to the heterogeneity of the fraction 1, anomeric configuration of the QuiNAc could not be established for subsequent O-specific polysaccharide repeating units. The study supported significant variability of *P. shigelloides* O-specific polysaccharides structures presented by 102 O-serotypes, pointing out the core oligosaccharides as possible targets for developed active and passive immunisation.

Moreover, a new core oligosaccharide composed of an undecasaccharide is reported herein, which represents the new core type among known *P. shigelloides* LPS. It is characterised by some heterogeneity corresponding to the absence of the terminal β-d-Glc*p* residue, that is characteristic for core oligosaccharides of *P. shigelloides*. Minor glycoforms represented the complete core OS with one additional β-d-Qui*p*NAc residue and a nonstoichiometric Gly substitution. The presence of the core OS glycoform substituted by the terminal Qui*p*NAc cannot be explained by the in-source OSRU fragmentation (Figure 3b), thus its origin cannot be explained based on the collected data. This new type of core OS shares features that are typical for the majority of *P. shigelloides* core regions: an inner region consisting of GalA, Glc, Gal, Kdo, and three Hep residues. Characteristic lack of phosphate groups in the core oligosaccharide structure distinguished it from the known LPS structure of bacteria from the *Enterobacteriaceae* family. Phosphate groups are very important carriers of negative charges, playing an important role in stabilising the conformation of the entire LPS molecule, and thus the bacterial membrane, by interacting with doubly charged Ca^2+^ and Mg^2+^ ions. The role of negatively charged residues in the investigated core oligosaccharide is played by GalA instead.

The core OS of *P. shigelloides* 90/89 is similar to the previously reported core oligosaccharide of *P. shigelloides* strain CNCTC 78/89 [22], differing only by one sugar residue in the outer core, that is the 6-substituted Glc instead of the 6-substituted GlcN. In the core OS of *P. shigelloides* O33 [20], both glycoforms, containing 6-substituted GlcN or 6-substituted Glc and O-acetylation of the terminal GalNAc, were reported. The core OS of *P. shigelloides* 90/89 is also similar to the core OS of serotype O33, that contained the 6-substituted Glc, but was devoid of O-acetyl.

The results presented herein broaden the knowledge about *P. shigellodes* lipopolysaccharides and may form the basis for further research, to clarify the relationship between the structure and biological activity of endotoxins. Structural analysis is the first step in understanding the unusual physicochemical properties of LPS, which can be used in the future research on the relationships between the structure of LPS and the type of aggregates formed in the aquatic environment, which may have an impact on the pathogenicity of *P. shigelloides* bacteria.

## 4. Materials and Methods

### 4.1. Bacteria

*P. shigelloides* serovar O22:H3 (strain CNCTC 90/89) was obtained from the collection of the Institute of Hygiene and Epidemiology, Prague, Czech Republic. Bacteria were grown and harvested as described previously [22].

### 4.2. Lipopolysaccharide and Oligosaccharides OS and OSRU

LPS was extracted from bacterial cells by the hot phenol/water method [31] and purified as previously reported [32]. Both phenol and water phases were collected and dialysed extensively against de-ionised water and purified by ultracentrifugation. The LPS obtained from the water phase (200 mg) was hydrolysed with 1.5% acetic acid at 100 °C for 30 min. The reaction mixture was freeze–thaw cycled, and then incubated at 100 °C for 15 min, and finally centrifuged (40,000× *g*, 20 min). The core OS (43.8 mg, average yield) was separated from core oligosaccharides substituted with one repeating unit of the O-antigen (19.48 mg, average yield) by size-exclusion chromatography, on a Bio-Gel P-10 column (1.6 × 100 cm) equilibrated with 0.05 M pyridine/acetic acid buffer of pH 5.6. A Knauer differential refractometer was used for monitoring of eluates. After, all fractions were freeze-dried and checked by ^1^H NMR spectroscopy and MALDI-TOF mass spectrometry.

### 4.3. Partial Acid Hydrolysis

A sample of the O-specific polysaccharide (fraction 1) was hydrolysed with 48% hydrofluoric acidat −20 °C. Progress of hydrolysis was checked by MALDI-TOF MS. The products obtained by hydrolysis after 24 h were lyophilised for further MALDI-TOF MS analysis.

### 4.4. Analytical Methods

Monosaccharides were examined as their alditol acetates by GC-MS [32]. Partially methylated alditol acetates were analysed according to the method of Ciucanu and Kerek [43] by GC-MS using a Thermo Scientific ITQ system using a ZebronTM ZB-5HT (Thermo Fisher Scientific, Waltham, MA, USA) GC Capillary Column (30 m × 0.25 mm × 0.25 μm), and with temperature program gradient from 150 to 270 °C at 8 °C/min. The absolute configurations of the monosaccharides were determined using (−)-2-butanol for the formation of 2-butyl glycosides [44,45].

### 4.5. NMR Spectroscopy

NMR spectra of OS and OSRU were obtained on a Bruker 600 MHz spectrometer. The sample was first repeatedly exchanged with D_2_O (99%) with intermediate lyophilisation. NMR spectra were obtained for D_2_O solutions or 9:1 D_2_O:H_2_O mixture (for identification of the exchangeable protons of the amide groups) at 25 °C. Internal calibration was applied using acetone (2.225 ppm, 31.05 ppm). The NMR signals were assigned by one- and two-dimensional experiments (COSY, TOCSY, NOESY, HMBC, HSQC-TOCSY, and HSQC-DEPT). The *J*_C-1,H-1_ constant values were achieved from a non-decoupled HSQC-DEPT experiment. The TOCSY experiments were recorded with the mixing times 30, 60, and 100 ms. The delay time in the HMBC experiment was set to 60 ms and the mixing time in the NOESY experiment was 200 ms. For observation of phosphate groups, a two-dimensional (2D) ^1^H,^31^P HMBC NMR spectrum was recorded. The spectra were acquired and processed with the help of standard Bruker software (Bruker BioSpin GmbH, Rheinstetten, Germany). The 2D spectra were assigned using the NMRFAM-SPARKY program [46].

### 4.6. Mass Spectrometry

The MALDI-TOF MS spectra were obtained on an Ultraflextreme (Bruker, Bremen, Germany) instrument in a positive ion mode. Samples were dissolved in water (1 mg/mL). The 2,5-Dihydroxybenzoic acid (10 mg/mL in 1:1 AcN/0.2 M citric acid [*v/v*]) was used as a matrix for analyses. For MALDI TOF/TOF MS analysis using the LIFT mode, the ion source voltage was set at 7.5 kV with a precursor ion mass window of 4 Da. The precursor ion was accelerated at 19.0 kV in the LIFT cell. The reflector voltage was set at 29.5 kV. External calibration was applied using the Peptide Calibration Standard II (Bruker Daltonics, Germany). Ions were interpreted according to the nomenclature of Domon and Costello [36].

## 5. Conclusions

Structural studies of *P. shigelloides* 90/89 lipopolysaccharide have been presented. This study reported the structure of the pentasaccharide repeating unit constituting the O-specific polysaccharide of *P. shigelloides* CNCTC 90/89 (O22) LPS. It contains rare monosaccharides such as β-d-Qui*p*NAc and four Gal*p*NAcAN residues. Moreover, a new type of the core oligosaccharide composed of an undecasaccharide was presented, that shares common *P. shigelloides* features: the lack of phosphate groups and the presence of uronic acids. 

## Figures and Tables

**Table 1:** The ^1^H and ^13^C NMR chemical shifts and selected inter-residue correlations from NOESY and HMBC spectra of the OS and OSRU fractions isolated from *P. shigelloides* 90/89 LPS.

Residue ^(a)^		Fraction ^(b)^		Chemical Shifts (ppm) ^(c)^								Selected Inter-Residue NOE and ^3^*J*_H,C_ Connectivities	
		OS	OSRU	H1 C1	H2, (H3′) C2	H3 C3	H4 C4	H5 C5	H6, H6′ C6	H7, H7′ C7	H8, H8′ C8	H1/C1 Connectivities to	Inter-Residue Atom/Residue
**A**	→5)-α-d-Kdo*p*	*	*		(2.26)	1.87 34.3	4.13 66.5	4.13 75.5	3.70 70.0	3.87 72.1	3.60, 3.84 64.2		
B	→4)-α-d-Gal*p*A-(1→	*	*	5.34 102.2	3.84 69.2	4.25 69.2	4.39 80.4	4.60 72.3	174.6			4.01 82.0	H3 of **C**
B′	→4)-α-d-Gal*p*A-(1→	*	*	5.43 102.6	3.85 69.1	4.25 69.1	4.42 80.6	4.67 72.4	174.3				
C	→3,7)-l-α-d-Hep*p*-(1→	*	*	5.29 102.2	4.20 70.7	4.01 82.0	4.02 65.5	3.61 73.4	4.15 69.1	3.50, 3.97 73.9		4.14	H3 of **E**
C′	→2,3,7)-l-α-d-Hep*p*-(1→ ^(d)^	*	*	5.39 100.0	4.23 79.1	nd	nd	nd	nd	nd			
D	α-d-Gal*p*N-(1→	*	*	5.29 94.9	3.40 51.9	4.11 67.1	4.01 68.8	4.30 72.0	3.72 61.7			4.57 76.5	H4 of **F**
E	→3,4)-l-α-d-Hep*p*-(1→	*	*	5.13 101.5	4.04 71.2	4.14 75.6	4.24 75.2	4.18 72.0	4.19 69.1	3.73 63.9		4.13 75.5	H5 of **A**
F	→4)-α-d-Gal*p*A-(1→	*	*	5.02 99.7	3.97 68.9	4.08 69.9	4.57 76.5	4.31 70.3	176.8			3.58	H7 of **I**
G	→6)-α-d-Glc*p*-(1→	*	*	4.98 100.9	3.40 72.6	3.69 74.0	3.55 69.7	4.31 72.0	3.60, 4.01 66.0			4.39 80.4	H4 of **B**
H	→4)-α-d-Gal*p*NAc-(1→	(*)	*	4.90 98.0	4.08 50.8	4.06 68.5	4.15 76.4	3.97 71.4	3.74 62.1			3.60 66.0	H6 of **G**
H	α-d-Gal*p*NAc-(1→	*		4.95 98.1	4.18 50.8	3.94 68.6	3.99 69.3	3.96 71.9	3.75 61.9			3.60 66.0	H6 of **G**
I	→7)-l-α-d-Hep*p*-(1→	*	*	4.88 103.6	3.96 71.1	3.86 71.3	3.90 66.8	3.61 73.3	4.23 68.5	3.58, 3.83 72.1		3.50	H7 of **C**
J	β-d-Gal*p*-(1→	*	*	4.54 104.5	3.50 72.3	3.68 73.2	3.91 69.7	3.66 76.0	3.69, 3.77 62.5			4.24 75.2	H4 of **E**
K	→3)-β-d-Qui*p*NAc-(1→		*	4.74 102.3	3.76 55.6	3.70 80.6	3.42 77.2	3.42 72.5	1.30 17.3			4.15	H4 of **H**
K	β-d-Qui*p*NAc-(1→	(*)		4.72 102.5	3.72 56.8	3.50 74.6	3.22 76.1	3.45 72.6	1.31 17.5			4.15	H4 of **H**
L	β-d-Glc*p*-(1→	*	*	4.59 103.7	3.23 74.1	3.50 75.5	3.47 69.9	3.59 76.2	3.79, 3.89 61.4			4.23 79.1	H2 of **C′**
M	→4)-α-d-Gal*p*NAcAN-(1→		*	5.41 99.0	4.24 50.0	4.03 67.0	4.45 76.1	4.26 71.2	174.4			3.70 80.6	H3 of **K**
N	→4)-α-d-Gal*p*NAcAN-(1→		*	5.07 98.4	4.22 50.0	4.17 67.1	4.44 76.3	4.85 71.4	174.5			4.46	H4 of **P**
O	α-d-Gal*p*NAcAN-(1→		*	5.04 98.6	4.16 50.1	4.05 67.8	4.28 69.6	4.86 72.1	175.1			4.44	H4 of **N**
P	→4)-α-d-Gal*p*NAcAN-(1→		*	5.08 98.4	4.23 50.0	4.22 67.0	4.46 76.0	4.88 71.4	174.0			4.45	H4 of **M**
Gly		*	*	3.98 40.9	169.0								

^(a)^ The *J*_C-1,H-1_ constants 179, 178, 177, 173, 174, 173, 176, 175, 161, 169, 161, 179, 178, 177, and 178 Hz were observed for B, C, D, E, F, G, H, I, J, K, L, M, N, O, and P residues, respectively. ^(b)^ Symbol * indicates the presence of the residue in the OS/OSRU and (*) indicates heterogeneity of the core OS attributed to nonstoichiometric substitution of the →4)-α-d-Gal*p*NAc-(1→ (H) by the β-d-Qui*p*NAc (K) (31%). ^(c)^ Average chemical shift values are shown for OS residues present as constituents of both OS and OSRU fractions. ^(d)^ Residue →2,3,7)-l-α-d-Hep*p*-(1→ was identified by methylation analysis, nd—not determined.

**Table 2:** Interpretation of positive ion mode MALDI-TOF mass spectra of the OS (Figure 3a) and the OSRU (Figure 3b) fractions isolated from LPS of *P. shigelloides* 90/89.

Oligosaccharide Structure ^a^	Calculated Mass (Da)	Observed Ion (*m/z*)	Calculated Ion (*m/z*)	Interpretation of the Ion
GalNAcAN_4_·QuiNAc·GalNAc·GalN·GalA_2_·Hex_3_·Hep_3_·Kdo·Gly	3125.03	3147.91	3148.02	[M+H, Na]^+^
GalNAcAN_4_·QuiNAc·GalNAc·GalN·GalA_2_·Hex_3_·Hep_3_·Kdo·Gly	3125.03	3129.91	3130.01	[M-H_2_O+H, Na]^+^
GalNAcAN_4_·QuiNAc·GalNAc·GalN·GalA_2_·Hex_3_·Hep_3_·Kdo·Gly	3125.03	3107.92	3108.03	[M-H_2_O+H]^+^
GalNAcAN_4_·QuiNAc·GalNAc·GalN·GalA_2_·Hex_3_·Hep_3_·Kdo	3068.01	3090.93	3091.00	[M+H, Na]^+^
GalNAcAN_4_·QuiNAc·GalNAc·GalN·GalA_2_·Hex_3_·Hep_3_·Kdo	3068.01	3068.95	3069.02	[M+H]^+^
GalNAcAN_4_·QuiNAc·GalNAc·GalN·GalA_2_·Hex_3_·Hep_3_·Kdo	3068.01	3050.94	3051.00	[M-H_2_O+H]^+^
GalNAcAN_4_·QuiNAc·GalNAc·GalN·GalA_2_·Hex_2_·Hep_3_·Kdo·Gly	2962.98	3007.91	3007.95	[M+H, 2Na]^+^
GalNAcAN_4_·QuiNAc·GalNAc·GalN·GalA_2_·Hex_2_·Hep_3_·Kdo·Gly	2962.98	2989.88	2989.94	[M-H_2_O+H, 2Na]^+^
GalNAcAN_4_·QuiNAc·GalNAc·GalN·GalA_2_·Hex_2_·Hep_3_·Kdo·Gly	2962.98	2967.92	2967.96	[M-H_2_O+H, Na]^+^
GalNAcAN_4_·QuiNAc·GalNAc·GalN·GalA_2_·Hex_2_·Hep_3_·Kdo·Gly	2962.98	2945.92	2945.97	[M-H_2_O+H]^+^
GalNAcAN_4_·QuiNAc·GalNAc·GalN·GalA_2_·Hex_2_·Hep_3_·Kdo	2905.96	2928.92	2928.95	[M+H, Na]^+^
GalNAcAN_4_·QuiNAc·GalNAc·GalN·GalA_2_·Hex_2_·Hep_3_·Kdo	2905.96	2906.93	2906.97	[M+H]^+^
GalNAcAN_4_·QuiNAc·GalNAc·GalN·GalA_2_·Hex_2_·Hep_3_·Kdo	2905.96	2888.92	2888.95	[M-H_2_O+H]^+^
QuiNAc·GalNAc·GalN·GalA_2_·Hex_3_·Hep_3_·Kdo·Gly	2260.73	2261.52	2261.74	[M+H]^+^
QuiNAc·GalNAc·GalN·GalA_2_·Hex_3_·Hep_3_·Kdo	2203.71	2226.47	2226.70	[M+H, Na]^+^
QuiNAc·GalNAc·GalN·GalA_2_·Hex_3_·Hep_3_·Kdo	2203.71	2204.47	2204.72	[M+H]^+^
QuiNAc·GalNAc·GalN·GalA_2_·Hex_3_·Hep_3_·Kdo	2203.71	2186.44	2186.71	[M-H_2_O+H]^+^
QuiNAc·GalNAc·GalN·GalA_2_·Hex_2_·Hep_3_·Kdo·Gly	2098.68	2099.38	2099.69	[M+H]^+^
QuiNAc·GalNAc·GalN·GalA_2_·Hex_2_·Hep_3_·Kdo	2041.66	2064.33	2064.65	[M+H, Na]^+^
GalNAc·GalN·GalA_2_·Hex_3_·Hep_3_·Kdo	2016.63	2039.28	2039.62	[M+H, Na]^+^
GalNAc·GalN·GalA_2_·Hex_3_·Hep_3_·Kdo	2016.63	2017.28	2017.64	[M+H]^+^
GalNAc·GalN·GalA_2_·Hex_3_·Hep_3_·Kdo	2016.63	1999.26	1999.62	[M-H_2_O+H]^+^
GalNAc·GalN·GalA_2_·Hex_2_·Hep_3_·Kdo·Gly	1911.60	1934.20	1934.59	[M+H, Na]^+^
GalNAc·GalN·GalA_2_·Hex_2_·Hep_3_·Kdo·Gly	1911.60	1912.20	1912.61	[M+H]^+^
GalNAc·GalN·GalA_2_·Hex_2_·Hep_3_·Kdo·Gly	1911.60	1894.18	1894.59	[M-H_2_O+H]^+^
GalNAc·GalN·GalA_2_·Hex_2_·Hep_3_·Kdo	1854.58	1877.16	1877.57	[M+H, Na]^+^
GalNAc·GalN·GalA_2_·Hex_2_·Hep_3_·Kdo	1854.58	1855.16	1855.58	[M+H]^+^
GalNAc·GalN·GalA_2_·Hex_2_·Hep_3_·Kdo	1854.58	1837.14	1837.57	[M-H_2_O+H]^+^

^a^ The Hex stands for t-Glc, t-Gal, and 6-substituted Glc residues in the structure, as shown in Figure 1a. The Gly stands for glycine residue.

**Table 3:** Interpretation of the positive ion mode MALDI-TOF mass spectrum of the partially hydrolysed O-specific polysaccharide fraction isolated from *P. shigelloides* 90/89 LPS (fraction 1).

Oligosaccharide Structure	Calculated Mass (Da)	Observed Ion (*m/z*)	Calculated Ion (*m/z*)	Interpretation of the Ion
GalNAcAN_4_·QuiNAc	1069.39	1074.39	1074.36	[M-H_2_O+H, Na]^+^
GalNAcAN_5_·QuiNAc	1285.47	1290.47	1290.43	[M-H_2_O+H, Na]^+^
GalNAcAN_6_·QuiNAc	1501.54	1506.56	1506.51	[M-H_2_O+H, Na]^+^
GalNAcAN_6_·QuiNAc	1501.54	1525.57	1525.54	[M+H, Na]^+^
GalNAcAN_7_·QuiNAc	1717.62	1722.61	1722.58	[M-H_2_O+H, Na]^+^
GalNAcAN_7_·QuiNAc	1717.62	1740.65	1740.61	[M+H, Na]^+^
GalNAcAN_8_·QuiNAc_2_	2120.78	2125.79	2125.74	[M-H_2_O+H, Na]^+^
GalNAcAN_9_·QuiNAc_2_	2336.85	2341.87	2341.82	[M-H_2_O +H, Na]^+^
GalNAcAN_10_·QuiNAc_2_	2552.93	2557.91	2557.89	[M-H_2_O+H, Na]^+^
GalNAcAN_10_·QuiNAc_2_	2552.93	2576.93	2576.92	[M+H, Na]^+^
GalNAcAN_11_·QuiNAc_2_	2769.00	2792.02	2791.99	[M+H, Na]^+^
GalNAcAN_12_·QuiNAc_3_	3172.16	3195.20	3195.14	[M+H, Na]^+^
GalNAcAN_13_·QuiNAc_3_	3388.23	3411.31	3411.22	[M+H, Na]^+^
GalNAcAN_14_·QuiNAc_3_	3604.31	3627.43	3627.30	[M+H, Na]^+^
GalNAcAN_15_·QuiNAc_3_	3820.38	3843.58	3843.37	[M+H, Na]^+^

## Abbreviations

LPS	lipopolysaccharide
OS	oligosaccharide
OSRU	core oligosaccharide substituted by one repeating unit of O-specific polysaccharide
MALDI-TOF	matrix-assisted laser-desorption/ionisation time-of-flight
MS	mass spectrometry
NMR	nuclear magnetic resonance
COSY	correlated spectroscopy
TOCSY	total correlation spectroscopy
NOESY	nuclear Overhauser effect spectroscopy
HMBC	heteronuclear multiple bond correlation
HSQC	heteronuclear single quantum coherence
DEPT	distortionless enhancement by polarisation transfer
